# Supplementary material for: Nonlinear association between C-reactive protein and severity of diabetic foot infection in patients with diabetes: A retrospective cohort study with clinical implications
Source: PLoS One. 2025 May 15;20(5):e0323704. doi: 10.1371/journal.pone.0323704 (PMC12080835; doi:10.1371/journal.pone.0323704)
Supplement: S1 Table — Adjustment factors included Sex, Age, Smoking, Diabetes duration, Hypertension, coronary heart disease, Cerebral infarction, BMI, MAP. (DOCX) [file pone.0323704.s001.docx]

Table S1.Threshold effect analysis of C-reactive protein on Severity of diabetic foot infection

| **C-reactive protein** | **OR** | **95%CI** | **P value** |
| --- | --- | --- | --- |
| **＜105mg/L** | 1.039 | 1.019-1.06 | ＜0.001 |
| **≥105mg/L** | 1.006 | 0.998-1.015 | 0.1307 |

Adjustment factors included Sex，Age，Smoking, Diabetes duration , Hypertension, coronary heart disease, Cerebral infarction, BMI，MAP
