# Supplementary material for: Nonlinear association between C-reactive protein and severity of diabetic foot infection in patients with diabetes: A retrospective cohort study with clinical implications
Source: PLoS One. 2025 May 15;20(5):e0323704. doi: 10.1371/journal.pone.0323704 (PMC12080835; doi:10.1371/journal.pone.0323704)
Supplement: Attachment — Submitted filename: Response to Reviewers.docx [file pone.0323704.s004.docx]

**Responds to the reviewer and editor’s comments:**

**Reply to Academic Editor:**

**Comments:**

1. Please ensure that your manuscript meets PLOS ONE's style requirements, including those for file naming. The PLOS ONE style templates can be found at

https://journals.plos.org/plosone/s/file?id=wjVg/PLOSOne_formatting_sample_main_body.pdf and

<https://journals.plos.org/plosone/s/file?id=ba62/PLOSOne_formatting_sample_title_authors_affiliations.pdf>

2. Thank you for stating the following financial disclosure:
“Taiyuan Bureau of Science and Technology,Science, Technology, and Innovation Program of National Regional Medical Center （202210”
Please state what role the funders took in the study. If the funders had no role, please state: "The funders had no role in study design, data collection and analysis, decision to publish, or preparation of the manuscript."
If this statement is not correct you must amend it as needed.
Please include this amended Role of Funder statement in your cover letter; we will change the online submission form on your behalf.
3. In the online submission form, you indicated that [The data underlying this article will be shared by the corresponding author upon reasonable request.].All PLOS journals now require all data underlying the findings described in their manuscript to be freely available to other researchers, either 1. In a public repository, 2. Within the manuscript itself, or 3. Uploaded as supplementary information.This policy applies to all data except where public deposition would breach compliance with the protocol approved by your research ethics board. If your data cannot be made publicly available for ethical or legal reasons (e.g., public availability would compromise patient privacy), please explain your reasons on resubmission and your exemption request will be escalated for approval.
4.  Please amend your manuscript to include your abstract after the title page.
5. [Please include captions for your Supporting Information files at the end of your manuscript, and update any in-text citations to match accordingly. Please see our Supporting Information guidelines for more information: http://journals.plos.org/plosone/s/supporting-information.](https://www.editorialmanager.com/pone/Please%20include%20captions%20for%20your%20Supporting%20Information%20files%20at%20the%20end%20of%20your%20manuscript,%20and%20update%20any%20in-text%20citations%20to%20match%20accordingly.%20Please%20see%20our%20Supporting%20Information%20guidelines%20for%20more%20information:%20http:/journals.plos.org/plosone/s/supporting-information.%20)

**Comment 1:**Please ensure that your manuscript meets PLOS ONE's style requirements, including those for file naming. The PLOS ONE style templates can be found at

https://journals.plos.org/plosone/s/file?id=wjVg/PLOSOne_formatting_sample_main_body.pdf and

<https://journals.plos.org/plosone/s/file?id=ba62/PLOSOne_formatting_sample_title_authors_affiliations.pdf>

**Response 1:**

Thank you for the valuable suggestions. Our manuscript has been revised to meet PLOS ONE's style requirements, including file naming.

**Comment 2:**Thank you for stating the following financial disclosure:
“Taiyuan Bureau of Science and Technology,Science, Technology, and Innovation Program of National Regional Medical Center （202210”
Please state what role the funders took in the study. If the funders had no role, please state: "The funders had no role in study design, data collection and analysis, decision to publish, or preparation of the manuscript."
If this statement is not correct you must amend it as needed.
Please include this amended Role of Funder statement in your cover letter; we will change the online submission form on your behalf.

**Response 2:**

Role of Funder statement

The funders had a role in the decision to publish or in the review and editing of the manuscript.And we had included this revised Role of Funder statement in a cover letter.

**Comment3:**In the online submission form, you indicated that [The data underlying this article will be shared by the corresponding author upon reasonable request.].All PLOS journals now require all data underlying the findings described in their manuscript to be freely available to other researchers, either 1. In a public repository, 2. Within the manuscript itself, or 3. Uploaded as supplementary information.This policy applies to all data except where public deposition would breach compliance with the protocol approved by your research ethics board. If your data cannot be made publicly available for ethical or legal reasons (e.g., public availability would compromise patient privacy), please explain your reasons on resubmission and your exemption request will be escalated for approval.

**Response 3:**

All data underlying the results of this study are available in the manuscript.

**Comment 4:** Please amend your manuscript to include your abstract after the title page.

**Response 4:**

An abstract has been added after the title page of the revised manuscript.

**Comment 5:**[Please include captions for your Supporting Information files at the end of your manuscript, and update any in-text citations to match accordingly. Please see our Supporting Information guidelines for more information: http://journals.plos.org/plosone/s/supporting-information.](https://www.editorialmanager.com/pone/Please%20include%20captions%20for%20your%20Supporting%20Information%20files%20at%20the%20end%20of%20your%20manuscript,%20and%20update%20any%20in-text%20citations%20to%20match%20accordingly.%20Please%20see%20our%20Supporting%20Information%20guidelines%20for%20more%20information:%20http:/journals.plos.org/plosone/s/supporting-information.%20)

**Response 5:**

The title of the supporting information document has been added at the end of the manuscript and the in-text citations have been updated accordingly.

**Reply to Additional Editor Comments:**

**Comment :**1.The author should give descriptions about the inclusion and exclusion for study population selection.
2. How do you identify the sample size? You may give some calculation process.
3. For covariates, each covariate should give a specific definition.
4 for laboratory parameters, the examination methods should be descripted.
5. For Table 1, the decimal places should be consistent
6. The study population were divided into three groups according to the C-reactive, how do you identify the cut point? And Is there no population less than 0.62?
7. Some study limitations should be addressed in the discussion.

**Comment 1:**1.The author should give descriptions about the inclusion and exclusion for study population selection.

**Response 1:**

Thank you for the detailed review. The inclusion and exclusion criteria for the selection of study participants are restated in the Methods section,, paragraph 1.Page 4, line 30-37.

In this retrospective cohort study, we reviewed the medical records of patients with diabetic foot infections at Taiyuan Central Hospital from January 2020 to September 2023.During the study period，406 patients were diagnosed with diabetic foot. After excluding patients with Wagner stage 2 and below, cancer and patients with incomplete data. And because the study population was patients with diabetes-related foot ulcers, other inflammatory causes of foot breakdown such as Buerger's disease, inflammatory bowel disease, and rheumatic diseases in diabetic patients were also excluded. A total of 301 patients with Wagner stage 3 or higher diabetes-related foot ulcers combined with moderate to severe infections were included in the study (Fig 1).Fig. 1 Flowchart of the patient selection process.

**Comment 2:** 2.How do you identify the sample size? You may give some calculation process.
**Response 2:**

Thank you for the detailed review. The sample size and some calculations for the selection of study participants have been added in the Methods section, paragraph 2.Page 4, lines 38-41.

This was a cross-sectional study and based on the access to the incidence of diabetic foot infection was 15% ^1^with a tolerance error of 0.03, a two-sided test was set up with a=0.05, and the sample size was calculated to be196.Considering the 20% loss to follow-up rate, a minimum of 236 patients with diabetic foot infection would need to be investigated.

**Comment 3:**3. For covariates, each covariate should give a specific definition.
4 for laboratory parameters, the examination methods should be descripted.

**Response 3:**

Thank you again for your valuable suggestions to improve the quality of our manuscript. Covariates, specific definition and laboratory parameters, the methods of investigation are restated in the Methods section, paragraph 3,4.Page 3, lines 43-63.

**Comment 4:** 5.For Table 1, the decimal places should be consistent

**Response 4:**

Thanks for your suggestion. We have adjusted the number of decimal places in Table 1 to be consistent.

**Comment 5:**6. The study population were divided into three groups according to the C-reactive, how do you identify the cut point? And Is there no population less than 0.62?

**Response 5:**

Thanks for your suggestion. Studies^2–4^have proposed the use of the CRP as a diagnostic cut-off for the disease, but there is no clear harmonisation of the cut-off for this indicator. The use of tertile grouping of C-reactive protein in this study allows for a relatively balanced population between groups and may explore some new findings.

We have added the relevant content in the Statistical analysis section,paragraph 1.Page 6, lines 70-76.

**Comment 6:**7. Some study limitations should be addressed in the discussion.

**Response 6:**

We think this is an excellent suggestion. We have been added the limitations of the study as below. Page 12-13, line 168-174.

This study had some limitations that need to be noted. First, although we excluded some inflammatory conditions that may affect CRP levels, we cannot exclude the possibility that CRP may be affected by other inflammatory conditions. Second, as with all observational studies, selection bias may have occurred. Thirt,t he study population is from a single institution, which may limit generalisability. Finally,t he wide 95% confidence intervals for the correlation between CRP and DFI severity may be related to sample size, population heterogeneity, and the non-specific nature of CRP as a marker of inflammation, necessitating larger standardised studies with multi-biomarker validation to improve clinical interpretability.

**Reply to Reviewer #1**

Dear Reviewer,

Thank you very much for your time in reviewing the manuscript and for your great suggestion to improve the accessibility of our manuscript.

**Comments:**

Reviewer #1: This study aimed to examine the association between CRP levels and the severity of diabetic foot infections (DFIs) in patients with diabetes using a retrospective "cohort" design and claiming to employ techniques such as generalized additive models and two-piecewise linear regression.
However, the manuscript is laden with extensive reporting flaws and deficiencies, compounded by poor language quality and numerous linguistic errors. These issues render a meaningful scientific review or extraction of valid science from the manuscript in its current state largely impossible. The authors are strongly advised to rewrite the entire manuscript in adherence to an appropriate reporting guideline (e.g., STROBE) and to seek comprehensive language editing by a professional scientific editor specializing in English.

**Response :**

Thanks for your great suggestion on improving the accessibility of our manuscript.We haved rewrited the entire manuscript in adherence to an appropriate reporting guideline and to re-seek comprehensive language editing by a professional scientific editor specializing in English.

**Reply to Reviewer #2**

Dear Reviewer,

Thank you very much for your time involved in reviewing the manuscript and your very encouraging comments on the merits.

**Comments:**

General recommendations:
**The title of the article is clear but could be improved by adding a phrase to emphasize its clinical relevance. For example, "Nonlinear Association Between C-Reactive Protein and Severity of Diabetic Foot Infection in Patients with Diabetes: A Retrospective Cohort Study with Clinical Implications" would make the study’s impact more evident.
The abstract is generally well-structured, providing key information on the background, methods, results, and conclusions. However, the introduction could benefit from a more detailed explanation of the need for this review, along with a general overview at the beginning. It could mention the lack of similar studies and their implications for clinical practice. In the methods section, clarify the study period covered and specify the databases searched. In the results section, highlight the strongest associated factors, and the conclusion could include a more specific clinical recommendation, such as the need to integrate CRP levels into diagnostic algorithms for DFI severity.
**In the introduction, there is a good explanation of diabetic foot ulcers (DFUs) as a public health issue. It would be helpful to include more details on the global burden of DFUs and comparisons with other diabetes-related complications. When mentioning previous studies, briefly summarize their findings on CRP and DFI to emphasize the variability in results and the need for this study. The introduction could also more explicitly highlight the novelty of this review compared to existing studies, particularly in exploring the nonlinear relationship between CRP and DFI severity.
**The methods section is thorough and follows standard guidelines for retrospective cohort studies. In the search strategy, consider providing more details on the rationale for choosing specific covariates and how CRP tertiles were determined. The inclusion and exclusion criteria are mostly clear, but you could elaborate on the reasons for excluding patients with other inflammatory conditions or incomplete medical records. It would also be helpful to expand on the statistical rationale for using a two-piecewise linear regression model.
**The results section is well-organized, and the quantitative analysis is robust. However, the data presentation could be more engaging by including a brief narrative summary before diving into the statistical details. For example, introduce the study characteristics and then transition into the statistical results. The figures, while informative, could be improved. Ensure that all figures are presented in high resolution (300 DPI or higher). Additionally, it would be useful to include a detailed explanation of significant findings in a more digestible format, such as simplified explanations of key associations and their implications.
*In the discussion, the findings are effectively related to previous research, but this section could be further strengthened by expanding comparisons with studies from other countries or regions with similar patient populations. Additionally, more explanation is needed on the mechanisms behind the observed threshold effect, such as why CRP levels below 105 mg/L show a different relationship with DFI severity compared to higher levels. Furthermore, the clinical implications of the findings should be expanded. Discuss specific interventions or policy recommendations, such as integrating CRP levels into diagnostic algorithms or developing targeted treatment strategies based on CRP thresholds.
Specific recommendations:
**Introduction
Some sentences are lengthy and could be simplified for better readability. For example:
"Neuropathy and vasculopathy underlie the pathology of diabetic foot ulcers, which in turn are complicated by infections, leading to significant risks such as amputation and even death."
**Materials and Methods
(Lines 35–39)
Grammatical error: "were included the research" should be "were included in the research."
Unclear phrasing: "in1 July 2023 format" is awkward and unclear.
Missing details: It would be helpful to briefly explain the eligibility criteria for inclusion/exclusion.
(Lines 41–46)
Minor grammatical issues: Missing spaces after commas in some places (e.g., "pressure,smoking").
The list of covariates is comprehensive but could be better organized for readability.
(Lines 48–56)
Grammatical errors: "was involved only the skin" should be "involving only the skin."
Inconsistent formatting: "And the ulcers" should not start with "And."
Missing clarity: The criteria for mild, moderate, and severe infections could be presented in a more structured format (e.g., bullet points or subheadings).
(Lines 58–61)
No major issues. This section is clear and concise.
(Lines 63–76)
Grammatical errors: "the demographic traits and clinical metrics was quantified" should be "were quantified."
Repetition: The two-piecewise linear regression model is described twice.
Clarity: The description of the statistical models could be more concise and structured.
**Results
(Lines 78–91)
Grammatical errors: "an average years of 65.2 ± 12.0 years" should be "an average age of 65.2 ± 12.0 years."
Unclear phrasing: "the participants comprised 208 (69.1%) male and 93 female" could be rephrased for clarity.
The sentence about differences among CRP tertiles is lengthy and could be broken into smaller sentences for readability.
Table 1. Baseline Characteristics
The table is well-structured but could benefit from clearer formatting and consistent use of symbols (e.g., "±" for standard deviation).
Some abbreviations (e.g., G+, G-) are not defined in the table legend.
(Lines 93–104)
Grammatical errors: "A high levels of CRP" should be "High levels of CRP."
Repetition: "The link endured" is vague and could be rephrased.
The sentence structure could be improved for clarity.
(Lines 106–112)
Grammatical errors: "After adjustment factors included" should be "After adjusting for factors including."
The description of the nonlinear relationship could be more concise.
(Lines 114–119)
The sentence structure could be improved for clarity.
"Produced uniform findings" is vague and could be rephrased.
**Discussion
The discussion is lengthy and could benefit from subheadings (e.g., "Comparison with Previous Studies," "Mechanistic Insights," "Clinical Implications") to improve readability.
The discussion does not explicitly address the limitations of the study. For example:
The retrospective design may introduce selection bias.
The study population is from a single institution, which may limit generalizability.
CRP levels can be influenced by other inflammatory conditions, which were not accounted for.
**Conclusion
The sentence "This relationship was associated with an increased DFI severity with increasing CRP when CRP was below 105 mg/L" is awkwardly phrased.
**Comment 1:**The title of the article is clear but could be improved by adding a phrase to emphasize its clinical relevance. For example, "Nonlinear Association Between C-Reactive Protein and Severity of Diabetic Foot Infection in Patients with Diabetes: A Retrospective Cohort Study with Clinical Implications" would make the study’s impact more evident.
**Response 1:**

Thank you for the valuable suggestions. This is an especially crucial issue. Based on your suggestions, we added our title’s expression into “ Nonlinear Association Between C-Reactive Protein and Severity of Diabetic Foot Infection in Patients with Diabetes: A Retrospective Cohort Study with Clinical Implications”. Page 1, line 1-3.

**Comment 2:**The abstract is generally well-structured, providing key information on the background, methods, results, and conclusions.

**Response 2:**

Thank you very much for your time involved in reviewing the manuscript and your very encouraging comments on the merits. We hope we can be better. Thank you.

**Comment 3:**However, the introduction could benefit from a more detailed explanation of the need for this review, along with a general overview at the beginning. It could mention the lack of similar studies and their implications for clinical practice.

**Response 3:**

Thanks for your great suggestion on improving the accessibility of our manuscript.

We have added the need for this review, the results of similar studies and their implications for clinical practice in the introduction section, paragraph 2.Page 4-5, line 10-27.

The International Working Group on the Diabetic Foot (IWGDF) guidelines provide recommendations for categorizing diabetic foot infections based on their severity. According to the IDSA and IWGDF criteria, severe diabetic foot infections must have localized infections deeper than the epidermis and subcutaneous tissue, as well as systemic inflammatory response syndrome (SIRS) symptoms.^5^When the Laboratory Risk Indicator for Necrotizing Fasciitis-based (LRINEC) score was applied to diabetic foot infections, it revealed a strong association with amputation and mortality.^6^The score is not specifically designed for diabetic foot infections, and its predictive value takes into account the inclusion of inflammatory markers such as C-reactive protein (CRP) and white blood cell count (WBC). However, the IWGDF guidelines only consider white blood cell counts greater than 12,000 or less than 4,000 cells/mm³ as inflammatory markers, which are also included in the SIRS criteria. Several studies have demonstrated that patients with severe diabetic foot infections (DFI) exhibit significantly higher erythrocyte sedimentation rates (ESR) and C-reactive protein (CRP) levels, as well as significantly lower albumin levels.^7–9^ Timely diagnosis and accurate assessment of the severity of DFI based on sensitive inflammatory biomarkers is critical, with considerable benefits for debridement procedures and antibiotic use to decrease the risk of amputation. However, whether the specific relationship between CRP and diabetic foot infection is linear or non-linear is not detailed in any of the current studies. The aim of this study was to investigate the relationship between CRP and the severity of diabetic foot infections (DFI) with Wagner 3 and above ulcers, in particular to explore the non-linear relationship between CRP and DFI severity.

**Comment 4:**In the methods section, clarify the study period covered and specify the databases searched.

**Response 4:**

Thanks for your great suggestion on improving the accessibility of our manuscript.

We have adapted the elements in the Study design and Participants section. Page 5, line 30-37.

In this retrospective cohort study, we reviewed the medical records of patients with diabetic foot infections at Taiyuan Central Hospital from January 2020 to September 2023.During the study period，406 patients were diagnosed with diabetic foot. After excluding patients with Wagner stage 2 and below, cancer and patients with incomplete data. And because the study population was patients with diabetes-related foot ulcers, other inflammatory causes of foot breakdown such as Buerger's disease, inflammatory bowel disease, and rheumatic diseases in diabetic patients were also excluded. A total of 301 patients with Wagner stage 3 or higher diabetes-related foot ulcers combined with moderate to severe infections were included in the study.(Fig 1).**Fig. 1 Flowchart of the patient selection process.**

**Comment 5:**In the results section, highlight the strongest associated factors, and the conclusion could include a more specific clinical recommendation, such as the need to integrate CRP levels into diagnostic algorithms for DFI severity.

**Response 5:**

We think this is an excellent suggestion. We have been mentioned in the conclusion as below. Page 13, line 154-163.

Our study illustrates a nonlinear relationship between CRP levels and DFI severity in patients with DFI, with an inflection point of roughly 105 mg/L. A significant positive correlation was observed between CRP levels and DFI severity at CRP concentrations below 105 mg/L, suggesting the importance of considering the inflammatory status in the early management of DFI. Thus, it is necessary to incorporate CRP levels into diagnostic algorithms for DFI severity, thereby guiding clinicians in developing targeted treatment strategies for patients with diabetic foot infections.

**Comment 6:**In the introduction, there is a good explanation of diabetes-related foot ulcers (DFUs) as a public health issue. It would be helpful to include more details on the global burden of DFUs and comparisons with other diabetes-related complications. When mentioning previous studies, briefly summarize their findings on CRP and DFI to emphasize the variability in results and the need for this study. The introduction could also more explicitly highlight the novelty of this review compared to existing studies, particularly in exploring the nonlinear relationship between CRP and DFI severity.

**Response 6:**

Thanks for your great suggestion on improving the accessibility of our manuscript. We have made additions to the introductory section based on the above suggestions, paragraph 1-2.Page 4-5, line 1-27.

In 2021, the International Diabetes Federation(IDF) notified that over 536.6 million adults global, accounting for more than 10.5% of the global adult population, are expected to have diabetes mellitus, increasing to 12.2% (783.2 million) in 2045^10^. Diabetes complications continue to increase worldwide, and foot ulcers are one of the most common diabetic complications. Patients with diabetic foot are at high risk of amputation, disability and death when co-infections occur ^1112^.Neuropathy and vasculopathy underlie the pathology of diabetes-related foot ulcers. Complicated infections in addition to diabetes-related foot ulcers can significantly increase serious risks such as amputation and even death^13,14^. These are huge economic health burdens for patients, countries, and the world.

The International Working Group on the Diabetic Foot (IWGDF) guidelines provide recommendations for categorizing diabetic foot infections based on their severity. According to the IDSA and IWGDF criteria, severe diabetic foot infections must have localized infections deeper than the epidermis and subcutaneous tissue, as well as systemic inflammatory response syndrome (SIRS) symptoms.^5^When the Laboratory Risk Indicator for Necrotizing Fasciitis-based (LRINEC) score was applied to diabetic foot infections, it revealed a strong association with amputation and mortality.^6^The score is not specifically designed for diabetic foot infections, and its predictive value takes into account the inclusion of inflammatory markers such as C-reactive protein (CRP) and white blood cell count (WBC). However, the IWGDF guidelines only consider white blood cell counts greater than 12,000 or less than 4,000 cells/mm³ as inflammatory markers, which are also included in the SIRS criteria. Several studies have demonstrated that patients with severe diabetic foot infections (DFI) exhibit significantly higher erythrocyte sedimentation rates (ESR) and C-reactive protein (CRP) levels, as well as significantly lower albumin levels.^7–9^ Timely diagnosis and accurate assessment of the severity of DFI based on sensitive inflammatory biomarkers is critical, with considerable benefits for debridement procedures and antibiotic use to decrease the risk of amputation. However, whether the specific relationship between CRP and diabetic foot infection is linear or non-linear is not detailed in any of the current studies. The aim of this study was to investigate the relationship between CRP and the severity of diabetic foot infections (DFI) with Wagner 3 and above ulcers, in particular to explore the non-linear relationship between CRP and DFI severity.

**Comment 7:**The methods section is thorough and follows standard guidelines for retrospective cohort studies. In the search strategy, consider providing more details on the rationale for choosing specific covariates and how CRP tertiles were determined. The inclusion and exclusion criteria are mostly clear, but you could elaborate on the reasons for excluding patients with other inflammatory conditions or incomplete medical records. It would also be helpful to expand on the statistical rationale for using a two-piecewise linear regression model.

**Response 7:**

Thanks for your great suggestion. We have added the elements in the Study design and Participants,Covariates, Identification of DFI severity and Statistical analysis section. Page5-8,line 29-63,69-91.

Study design and Participants

In this retrospective cohort study, we reviewed the medical records of patients with diabetic foot infections at Taiyuan Central Hospital from January 2020 to September 2023.During the study period，406 patients were diagnosed with diabetic foot. After excluding patients with Wagner stage 2 and below, cancer and patients with incomplete data. And because the study population was patients with diabetes-related foot ulcers, other inflammatory causes of foot breakdown such as Buerger's disease, inflammatory bowel disease, and rheumatic diseases in diabetic patients were also excluded. A total of 301 patients with Wagner stage 3 or higher diabetes-related foot ulcers combined with moderate to severe infections were included in the study (Fig 1).**Fig. 1 Flowchart of the patient selection process.**

This was a cross-sectional study and based on the access to the incidence of diabetic foot infection was 15% ^11^with a tolerance error of 0.03, a two-sided test was set up with a=0.05, and the sample size was calculated to be196.Considering the 20% loss to follow-up rate, a minimum of 236 patients with diabetic foot infection would need to be investigated.

Covariates

The following potential covariates were analysed in this study, including sex, age, body mass index (BMI) and smoking status, mean arterial pressure, hypertension, coronary heart disease, cerebrovascular disease, fasting plasma glucose, glycosylated haemoglobin, erythrocyte count, haemoglobin, white blood cell count, neutrophil percentage, platelet count, C-reactive protein (CRP), albumin, duration of diabetes, DFI microbial community, severity of diabetic foot infection, amputation status. Smoking status was categorized as current, former, or never smoker^12^. The BMI is the ratio of height to weight squared. Comorbidities(Hypertension, coronary heart disease, and cerebrovascular disease)was determined by documenting in the electronic medical record whether or not a physician has made a previous diagnosis. The DFI microbial community is the result of general bacterial cultures of localised tissue from affected foot ulcers. Amputations were classified as minor or major. Minor amputations were defined as those where the ankle was left intact, and major amputations were above the ankle^13^.

Identification of DFI severity

The International Working Group on the Diabetic Foot has expanded the criteria for diagnosing and evaluating the severity of DFI ^14^. 1) Mild infection : Diabetic foot ulcer with no systemic manifestations involving only the skin or subcutaneous tissue (not any deeper tissues) or any erythema that does not extend > 2 cm. 2) Moderate infection :The ulcers deep to bone and fascia with erythema extending > 2 cm and without systemic manifestations .3) Severe infection: The presence of two or more systemic symptoms, such as body temperature exceeding 38°C or falling below 36°C, a heart rate surpassing 90 beats/min, a respiratory rate higher than 20 breaths/min, a partial pressure CO2 level below 4.3 kPa (32 mmHg), a WBC count exceeding 12,000 or falling below 4,000 cells/mm^3^, or the presence of more than 10% immature (banded) leukocytes.

Statistical analysis

This study aimed to investigate the association between CRP and DFI severity. There is currently no established diagnostic threshold for C-reactive protein in clinical practice. Patients in this study were divided into three groups based on CRP quantiles to ensure a relatively balanced population between groups and to explore some new findings. Data are presented as mean ± standard deviation (SD) or median (interquartile range, IQR) for continuous variables, and as frequencies (percentages) for categorical variables. Logistic regression analyses was used to estimate the odds ratio (OR) and corresponding 95% CI for DFI severity according to serum CRP levels.

Both non-adjusted and multivariate­adjusted models were used. Variables were adjusted if they changed the odds ratio by at least 10% after being added to the model, and clinically significant variables were also adjusted. Model 1 was adjusted covariates including age and sex, and model 2 adjusted for variables from model 1 plus smoking, diabetes duration, hypertension, coronary heart disease, cerebral infarction, BMI, Mean arterial pressure. A multivariate-adjusted restricted cubic spline model was constructed to establish the OR curves at 3 knots to examine the possible nonlinear dose response association between CRP and DFI severity .We used a two-piece-wise logistic regression model with smoothing to analyze the association threshold between CRP and DFI severity after adjusting the variables in Model 3. The likelihood-ratio test and the bootstrap resampling method were used to determine inflection points.

In addition, it was determined whether the relationship between CRP and DFI was stable in different population groups. Subgroup analyses were performed according to age, sex, BMI, smoking status, hypertension, coronary heart disease and cerebrovascular disease.

The R statistical software (version 4.2.2, http://www.R-project.org, The R Foundation) and the Free Statistics Analysis Platform Version 1.9^15^ were employed to carry out all the analyses, and p value less than 0.05 represented statistically significance.

**Comment 8:**The results section is well-organized, and the quantitative analysis is robust. However, the data presentation could be more engaging by including a brief narrative summary before diving into the statistical details. For example, introduce the study characteristics and then transition into the statistical results. The figures, while informative, could be improved. Additionally, it would be useful to include a detailed explanation of significant findings in a more digestible format, such as simplified explanations of key associations and their implications.
**Response 8:**

Thank you again for your suggestions. We have improved the results section. Page 8-11, line 92-124.We have set the resolution of all images to 300 DPI.

Results

Baseline characteristics

Baseline characteristics of study participants by CRP category are summarised in Table 1.Of the 406 patients with DFI, 301 eligible individuals were included. The participants comprised 208 male and 93 female, with an average age of 65.2 ± 12.0 years, and 21.9% of the patients had severe DFIs. Differences were noted among the three groups in terms of the severity of DFI, FBG, RBC, hemoglobin, WBC, neutrophil percentage, platelet count, and albumin (all P < 0.05). Otherwise, the distribution of patient characteristics was similar ( P > 0.05).

Association between CRP and DFI severity

In the multivariable logistic regression analysis, the odds ratio (OR) of CRP (per 1mg/dl increase) were consistently significant in all three models (OR:1.01) (Table 2). After adjustment for all covariates, patients with CRP T3 showed a 43.12-fold increase in the DFI severity (OR = 43.12, 95%CI: 9.87~188.25, p <0.001, model 2)，compared to CRP T1.

Restricted cubic spline analysis was used to investigate the dose-response relationship between CRP and DFI severity. Figure 2 shows a non-linear relationship between CRP and DFI severity (Fig 2).In the two-piecewise regression models, the adjusted OR for severe diabetic foot infection was 1.039 (95% CI, 1.019-1.06; P＜0.001) in participants with CRP＜105mg/L, whereas there was no association between CRP and DFI severity in participants with CRP≥105mg/L (Table S1).**Fig 2.Nonlinear dose-response relationship between CRP and severity of diabetic foot infection.**

Subgroup analyses

Figure 3 shows the results of the subgroup analyses. In the subgroup analyses, for patients who were at advanced age ,BMI and smoking or with coexisting conditions (hypertension, coronary heart disease, and cerebrovascular disease), the associations between CRP and DFI severity were statistically significant, and no significant interaction was detected.**Fig.3 Stratified analyses of the association between CRP and severity of diabetic foot infection according to baseline characteristics.**

**Comment 9:***In the discussion, the findings are effectively related to previous research, but this section could be further strengthened by expanding comparisons with studies from other countries or regions with similar patient populations. Additionally, more explanation is needed on the mechanisms behind the observed threshold effect, such as why CRP levels below 105 mg/L show a different relationship with DFI severity compared to higher levels. Furthermore, the clinical implications of the findings should be expanded. Discuss specific interventions or policy recommendations, such as integrating CRP levels into diagnostic algorithms or developing targeted treatment strategies based on CRP thresholds.
**Response 9:**

Thank you again for your suggestions. We have improved the elements in the discussion section. Page 11-13, line 125-167.

Discussion

In this retrospective cohort study, CRP was independently associated with DFI severity and demonstrated an inverted L-shaped relationship, with an inflection point of almost 105 mg/L. We observed a positive association between CRP and DFI severity in patients with CRP below 105 mg/L. Both the stratified analyses showed that the relationship between CRP and DFI severity remained robust. To the best of our knowledge, this is the first report of a non-linear association between CRP and DFI severity.

**Comparison with Previous Studies**

A multitude of epidemiological research has substantiated the role of CRP associated with the intensity of diabetic foot infections. A study that included a retrospective cohort of 245 patients with DFI found an association between necrosis, serum albumin, erythrocyte sedimentation rate, CRP, and neutrophil-to-lymphocyte ratio values, and severe DFI ^10^.Furthermore, In a clinical investigation conducted in South Korean hospitals, which included 123 patients diagnosed with DFI, it was found that higher concentrations of procalcitonin and CRP correlated with increased severity of DFI. ^16^.Several studies have confirmed the strong relationship between DFI and serum CRP levels^2,10,17,18^, but no reports of a non-linear relationship between the two have been found. In our study population, we confirmed a positive correspondence between CRP levels and the severity of DFI. And there is an inverted L-shaped relationship between the two. In other words, serum CRP levels was associated with increased DFI severity when CRP was low than 105 mg/L, and there was no association between the two when CRP was above 105 mg/L.

**Mechanistic Insights**

It's pertinent to note that CRP is an acute-phase reactant synthesized within hepatocytes^19^. When infection occurs in patients with diabetes-related foot ulcers, plasma levels of CRP rise rapidly and dramatically, up to 1,000 times higher than normal^2,20^. Specific mechanisms may be that CRP is promptly produced by hepatocytes under the regulation of cytokines such as interleukin-6 and interleukin-1, in which upregulation occurs through the stimulation of gene expression by the STAT3, C/EBP, and NF-κB pathways^20^.CRP binds to phospholipoproteins of localised bacterial cells in diabetes-related foot ulcers or exerts an immune effect by activating the complement converting enzyme C3. In addition, CRP binds to phagocytes and platelet-activating factors to reduce the inflammatory response^2^.The results of this study recommend that CRP is not only a sign of inflammation but is also associated with inflammatory and systemic immune responses^20^.

**Clinical Implications**

Our study found a nonlinear relationship between CRP levels and DFI severity, which was not found in previous studies. In the early stages of severe infections, the immune system can be over-activated, producing large amounts of inflammation-associated factors such as CRP, calcitoninogen and IL-6. However, as the infection progresses, the immune cells may enter a state of exhaustion and the ability to produce inflammatory factors decreases or even reaches saturation, preventing further immune response. Hence，high CRP levels may indicate a more severe infection or complications. These findings highlight the importance of considering low and high CRP levels when assessing DFI severity and tailoring treatment strategies accordingly. Additional research is required to elucidate the fundamental mechanisms behind this non-linear relationship.

The study showed that CRP was not useful to distinguish between noninfected and mild IDFU, and is the most informative marker for differentiating more serious foot infections^17,21^. Our study population of patients with diabetes-related foot ulcers grade 3 and above with moderate to severe infections further confirms that CRP is associated with severe infections, which is consistent with the above findings.

**Comment 10:**Specific recommendations:
**Introduction
Some sentences are lengthy and could be simplified for better readability. For example:
"Neuropathy and vasculopathy underlie the pathology of diabetic foot ulcers, which in turn are complicated by infections, leading to significant risks such as amputation and even death."

**Response 10:**

Thank you again for your valuable suggestions to simplify our expressions. We simplified "Neuropathy and vasculopathy underlie the pathology of diabetes-related foot ulcers, which in turn are complicated by infections, leading to significant risks such as amputation and even death." for "Neuropathy and vasculopathy underlie the pathology of diabetes-related foot ulcers. Complicated infections in addition to diabetes-related foot ulcers can significantly increase serious risks such as amputation and even death. "in our manuscript. Page 4, line 6-8 .

**Comment 11:****Materials and Methods
(Lines 35–39)
Grammatical error: "were included the research" should be "were included in the research."
Unclear phrasing: "in1 July 2023 format" is awkward and unclear.
Missing details: It would be helpful to briefly explain the eligibility criteria for inclusion/exclusion.
**Response11:**

Thank you again for your valuable suggestions to simplify our expressions. We simplified "were included the research" should be "were included in the research." in our manuscript. We deleted the phrase "in1 July 2023 format". In response to other comments we have been presented as below.Page 5, line 30-37.

In this retrospective cohort study, we reviewed the medical records of patients with diabetic foot infections at Taiyuan Central Hospital from January 2020 to September 2023.During the study period，406 patients were diagnosed with diabetic foot. After excluding patients with Wagner stage 2 and below, cancer and patients with incomplete data. And because the study population was patients with diabetes-related foot ulcers, other inflammatory causes of foot breakdown such as Buerger's disease, inflammatory bowel disease, and rheumatic diseases in diabetic patients were also excluded. A total of 301 patients with Wagner stage 3 or higher diabetes-related foot ulcers combined with moderate to severe infections were included in the research.

**Comment 12:**(Lines 41–46)
Minor grammatical issues: Missing spaces after commas in some places (e.g., "pressure,smoking").
The list of covariates is comprehensive but could be better organized for readability.
**Response12:**

Thanks for your great suggestion on improving the accessibility of our manuscript. We have improved the elements of the Covariates section. Page6, line 42-53.

Covariates

The following potential covariates were analysed in this study, including sex, age, body mass index (BMI) and smoking status, mean arterial pressure, hypertension, coronary heart disease, cerebrovascular disease, fasting plasma glucose, glycosylated haemoglobin, erythrocyte count, haemoglobin, white blood cell count, neutrophil percentage, platelet count, C-reactive protein (CRP), albumin, duration of diabetes, DFI microbial community, severity of diabetic foot infection, amputation status. Smoking status was categorized as current, former, or never smoker^12^. The BMI is the ratio of height to weight squared. Comorbidities(Hypertension, coronary heart disease, and cerebrovascular disease)was determined by documenting in the electronic medical record whether or not a physician has made a previous diagnosis. The DFI microbial community is the result of general bacterial cultures of localised tissue from affected foot ulcers. Amputations were classified as minor or major. Minor amputations were defined as those where the ankle was left intact, and major amputations were above the ankle^13^.

**Comment 13:**(Lines 48–56)
Grammatical errors: "was involved only the skin" should be "involving only the skin."
Inconsistent formatting: "And the ulcers" should not start with "And."
Missing clarity: The criteria for mild, moderate, and severe infections could be presented in a more structured format (e.g., bullet points or subheadings).
**Response13:**

Thanks for your great suggestion on improving the accessibility of our manuscript. Thanks for your great suggestion on improving the accessibility of our manuscript. We have improved the elements of the Identification of DFI severity section.Page 6, line 54-63.

Identification of DFI severity

The International Working Group on the Diabetic Foot has expanded the criteria for diagnosing and evaluating the severity of DFI ^14^. 1) Mild infection : Diabetic foot ulcer with no systemic manifestations involving only the skin or subcutaneous tissue (not any deeper tissues) or any erythema that does not extend > 2 cm. 2) Moderate infection :The ulcers deep to bone and fascia with erythema extending > 2 cm and without systemic manifestations .3) Severe infection: The presence of two or more systemic symptoms, such as body temperature exceeding 38°C or falling below 36°C, a heart rate surpassing 90 beats/min, a respiratory rate higher than 20 breaths/min, a partial pressure CO2 level below 4.3 kPa (32 mmHg), a WBC count exceeding 12,000 or falling below 4,000 cells/mm^3^, or the presence of more than 10% immature (banded) leukocytes.

**Comment 14:**(Lines 58–61)
No major issues. This section is clear and concise.
(Lines 63–76)
Grammatical errors: "the demographic traits and clinical metrics was quantified" should be "were quantified."
Repetition: The two-piecewise linear regression model is described twice.
Clarity: The description of the statistical models could be more concise and structured.
**Response14:**

Thanks for your great suggestion on improving the accessibility of our manuscript. We have improved the elements of the Statistical analysis section.Page7-8, line 69-91.

Statistical analysis

This study aimed to investigate the association between CRP and DFI severity. There is currently no established diagnostic threshold for C-reactive protein in clinical practice. Patients in this study were divided into three groups based on CRP quantiles to ensure a relatively balanced population between groups and to explore some new findings. Data are presented as mean ± standard deviation (SD) or median (interquartile range, IQR) for continuous variables, and as frequencies (percentages) for categorical variables. Logistic regression analyses was used to estimate the odds ratio (OR) and corresponding 95% CI for DFI severity according to serum CRP levels.

Both non-adjusted and multivariate­adjusted models were used. Variables were adjusted if they changed the odds ratio by at least 10% after being added to the model, and clinically significant variables were also adjusted. Model 1 was adjusted covariates including age and sex, and model 2 adjusted for variables from model 1 plus smoking, diabetes duration, hypertension, coronary heart disease, cerebral infarction, BMI, Mean arterial pressure. A multivariate-adjusted restricted cubic spline model was constructed to establish the OR curves at 3 knots to examine the possible nonlinear dose response association between CRP and DFI severity .We used a two-piece-wise logistic regression model with smoothing to analyze the association threshold between CRP and DFI severity after adjusting the variables in Model 3. The likelihood-ratio test and the bootstrap resampling method were used to determine inflection points.

In addition, it was determined whether the relationship between CRP and DFI was stable in different population groups. Subgroup analyses were performed according to age, sex, BMI, smoking status, hypertension, coronary heart disease and cerebrovascular disease.

The R statistical software (version 4.2.2, http://www.R-project.org, The R Foundation) and the Free Statistics Analysis Platform Version 1.9^15^ were employed to carry out all the analyses, and p value less than 0.05 represented statistically significance.

**Comment 15:****Results
(Lines 78–91)
Grammatical errors: "an average years of 65.2 ± 12.0 years" should be "an average age of 65.2 ± 12.0 years."
Unclear phrasing: "the participants comprised 208 (69.1%) male and 93 female" could be rephrased for clarity.
The sentence about differences among CRP tertiles is lengthy and could be broken into smaller sentences for readability.
Table 1. Baseline Characteristics
The table is well-structured but could benefit from clearer formatting and consistent use of symbols (e.g., "±" for standard deviation).
Some abbreviations (e.g., G+, G-) are not defined in the table legend.
**Response15:**

Thanks for your great suggestion on improving the accessibility of our manuscript. We have improved the elements of the Baseline characteristics section. Page 8, line 93-99.

Baseline characteristics

Baseline characteristics of study participants by CRP category are summarised in Table 1.Of the 406 patients with DFI, 301 eligible individuals were included. The participants comprised 208 male and 93 female, with an average age of 65.2 ± 12.0 years, and 21.9% of the patients had severe DFIs. Differences were noted among the three groups in terms of the severity of DFI, FBG, RBC, hemoglobin, WBC, neutrophil percentage, platelet count, and albumin (all P < 0.05). Otherwise, the distribution of patient characteristics was similar ( P > 0.05).

**Comment 16:**(Lines 93–104)
Grammatical errors: "A high levels of CRP" should be "High levels of CRP."
Repetition: "The link endured" is vague and could be rephrased.
The sentence structure could be improved for clarity.
**Response16:**

Thanks for your great suggestion on improving the accessibility of our manuscript. We have improved the elements of the Association between CRP and DFI severity section,paragraphs1-2. Page 10, line 104-114.

Association between CRP and DFI severity

In the multivariable logistic regression analysis, the odds ratio (OR) of CRP (per 1mg/dl increase) were consistently significant in all three models (OR:1.01) (Table 2). After adjustment for all covariates, patients with CRP T3 showed a 43.12-fold increase in the DFI severity (OR = 43.12, 95%CI: 9.87~188.25, p <0.001, model 2)，compared to CRP T1.

Restricted cubic spline analysis was used to investigate the dose-response relationship between CRP and DFI severity. Figure 2 shows a non-linear relationship between CRP and DFI severity (Fig 2).In the two-piecewise regression models, the adjusted OR for severe diabetic foot infection was 1.039 (95% CI, 1.019-1.06; P＜0.001) in participants with CRP＜105mg/L, whereas there was no association between CRP and DFI severity in participants with CRP≥105mg/L (Table S1).**Fig 2.Nonlinear dose-response relationship between CRP and severity of diabetic foot infection.**

**Comment 17:**(Lines 106–112)
Grammatical errors: "After adjustment factors included" should be "After adjusting for factors including."
The description of the nonlinear relationship could be more concise.
**Response17:**

Thanks for your great suggestion on improving the accessibility of our manuscript. We have improved the elements of Association between CRP and DFI severity in the Results section. Page 10, line 109-114.

Restricted cubic spline analysis was used to investigate the dose-response relationship between CRP and DFI severity. Figure 2 shows a non-linear relationship between CRP and DFI severity (Fig 2).In the two-piecewise regression models, the adjusted OR for severe diabetic foot infection was 1.039 (95% CI, 1.019-1.06; P＜0.001) in participants with CRP＜105mg/L, whereas there was no association between CRP and DFI severity in participants with CRP≥105mg/L (Table S1).**Fig 2.Nonlinear dose-response relationship between CRP and severity of diabetic foot infection.**

**Comment 18:**(Lines 114–119)
The sentence structure could be improved for clarity.
"Produced uniform findings" is vague and could be rephrased.
**Response18:**

Thanks for your great suggestion on improving the accessibility of our manuscript. We have improved the elements of Subgroup analyses in the Results section. Page 11, line 119-124.

Subgroup analyses

Figure 3 shows the results of the subgroup analyses. In the subgroup analyses, for patients who were at advanced age ,BMI and smoking or with coexisting conditions (hypertension, coronary heart disease, and cerebrovascular disease), the associations between CRP and DFI severity were statistically significant, and no significant interaction was detected.**Fig.3 Stratified analyses of the association between CRP and severity of diabetic foot infection according to baseline characteristics.**

**Comment 19:****Discussion
The discussion is lengthy and could benefit from subheadings (e.g., "Comparison with Previous Studies," "Mechanistic Insights," "Clinical Implications") to improve readability.
The discussion does not explicitly address the limitations of the study. For example:
The retrospective design may introduce selection bias.
The study population is from a single institution, which may limit generalizability.
CRP levels can be influenced by other inflammatory conditions, which were not accounted for.

**Response 19:**

Thanks for your great suggestion on improving the accessibility of our manuscript. We have revised the whole discussion section,Page 10-14, line 125-174.

Discussion

In this retrospective cohort study, CRP was independently associated with DFI severity and demonstrated an inverted L-shaped relationship, with an inflection point of almost 105 mg/L. We observed a positive association between CRP and DFI severity in patients with CRP below 105 mg/L. Both the stratified analyses showed that the relationship between CRP and DFI severity remained robust. To the best of our knowledge, this is the first report of a non-linear association between CRP and DFI severity.

**Comparison with Previous Studies**

A multitude of epidemiological research has substantiated the role of CRP associated with the intensity of diabetic foot infections. A study that included a retrospective cohort of 245 patients with DFI found an association between necrosis, serum albumin, erythrocyte sedimentation rate, CRP, and neutrophil-to-lymphocyte ratio values, and severe DFI ^10^.Furthermore, In a clinical investigation conducted in South Korean hospitals, which included 123 patients diagnosed with DFI, it was found that higher concentrations of procalcitonin and CRP correlated with increased severity of DFI. ^16^.Several studies have confirmed the strong relationship between DFI and serum CRP levels^2,10,17,18^, but no reports of a non-linear relationship between the two have been found. In our study population, we confirmed a positive correspondence between CRP levels and the severity of DFI. And there is an inverted L-shaped relationship between the two. In other words, serum CRP levels was associated with increased DFI severity when CRP was low than 105 mg/L, and there was no association between the two when CRP was above 105 mg/L.

**Mechanistic Insights**

It's pertinent to note that CRP is an acute-phase reactant synthesized within hepatocytes^19^. When infection occurs in patients with diabetes-related foot ulcers, plasma levels of CRP rise rapidly and dramatically, up to 1,000 times higher than normal^2,20^. Specific mechanisms may be that CRP is promptly produced by hepatocytes under the regulation of cytokines such as interleukin-6 and interleukin-1, in which upregulation occurs through the stimulation of gene expression by the STAT3, C/EBP, and NF-κB pathways^20^.CRP binds to phospholipoproteins of localised bacterial cells in diabetes-related foot ulcers or exerts an immune effect by activating the complement converting enzyme C3. In addition, CRP binds to phagocytes and platelet-activating factors to reduce the inflammatory response^2^.The results of this study recommend that CRP is not only a sign of inflammation but is also associated with inflammatory and systemic immune responses^20^.

**Clinical Implications**

Our study found a nonlinear relationship between CRP levels and DFI severity, which was not found in previous studies. In the early stages of severe infections, the immune system can be over-activated, producing large amounts of inflammation-associated factors such as CRP, calcitoninogen and IL-6. However, as the infection progresses, the immune cells may enter a state of exhaustion and the ability to produce inflammatory factors decreases or even reaches saturation, preventing further immune response. Hence，high CRP levels may indicate a more severe infection or complications. These findings highlight the importance of considering low and high CRP levels when assessing DFI severity and tailoring treatment strategies accordingly. Additional research is required to elucidate the fundamental mechanisms behind this non-linear relationship.

The study showed that CRP was not useful to distinguish between noninfected and mild IDFU, and is the most informative marker for differentiating more serious foot infections^17,21^. Our study population of patients with diabetes-related foot ulcers grade 3 and above with moderate to severe infections further confirms that CRP is associated with severe infections, which is consistent with the above findings.

This study had some limitations that need to be noted. First, although we excluded some inflammatory conditions that may affect CRP levels, we cannot exclude the possibility that CRP may be affected by other inflammatory conditions. Second, as with all observational studies, selection bias may have occurred. Thirt,t he study population is from a single institution, which may limit generalisability. Finally,t he wide 95% confidence intervals for the correlation between CRP and DFI severity may be related to sample size, population heterogeneity, and the non-specific nature of CRP as a marker of inflammation, necessitating larger standardised studies with multi-biomarker validation to improve clinical interpretability.

**Comment 20:****Conclusion
The sentence "This relationship was associated with an increased DFI severity with increasing CRP when CRP was below 105 mg/L" is awkwardly phrased.
**Response 20:**

Thanks for your great suggestion on improving the accessibility of our manuscript.

We substituted "This relationship was associated with an increased DFI severity with increasing CRP when CRP was below 105 mg/L" for "A significant positive correlation was observed between CRP levels and DFI severity at CRP concentrations below 105 mg/L "in our manuscript. Page 14, line 177-179 .

**Reply to Reviewer #3:**

Thank you very much for your time in reviewing the manuscript and for your great suggestion to improve the accessibility of our manuscript.

**Comments:**

The article reports results of a study to identify the association between C-reactive protein and severity of diabetic foot infection. This is a useful study to add to the current evidence on this association, however some areas of methods or text are unclear, as follows:
- Your abstract results report only 301 of the potential sample of 406 patients were included in the analysis. Some information on why over 100 patients were excluded is needed
- Section 2.1 - what are the inclusion and exclusion criteria for 'eligible individuals'? these need to be stated in full. the last sentence of this section needs a grammar edit.
- Section 2.2 - why were other comorbid conditions which may influence CRP levels not included as covariates? e.g. other infections, autoimmune or inflammatory conditions? This needs to be added to a section on limitations of the study
- p.4, paragraph before results - I'd suggest changing the wording of 'effect of CRP' as I don't believe the CRP effects DFI severity, instead it is associated with DFI severity?
- add very wide confidence intervals to the limitations section
- the article needs a language and grammar edit, e.g. first line of introduction 'million grown-up? do you mean adults?
- it is preferred to use the term diabetes-related foot ulcers, rather than diabetic foot ulcer.

**Comment 1:**Your abstract results report only 301 of the potential sample of 406 patients were included in the analysis. Some information on why over 100 patients were excluded is needed

**Response 1:**

Thanks for your great suggestion on improving the accessibility of our manuscript.

In the summary of abstract results section, we state that we "excluded some information from more than 100 patients". Page 2, line 39-45.

**Results**

Based on inclusion exclusion criteria，A total of 301 patients with Wagner stage 3 or higher diabetic foot ulcers combined with moderate to severe infections were included in the study at last. CRP levels were independently associated with the severity of DFIs after accounting for potential confounding factors. A nonlinear relationship was observed between CRP level and DFI severity, with a threshold of approximately 105 mg/L. The odds ratio for severe diabetic foot infection was 1.039 (95% confidence interval: 1.019–1.06, P < 0.001) in patients with DFI and CRP < 105 mg/L.

**Comment 2:**Section 2.1 - what are the inclusion and exclusion criteria for 'eligible individuals'? these need to be stated in full. the last sentence of this section needs a grammar edit.

**Response 2:**

Thanks for your great suggestion.We have added inclusion and exclusion criteria in Study design and Participants section of manuscript. Page 5, line 30-37.

Study design and Participants

In this retrospective cohort study, we reviewed the medical records of patients with diabetic foot infections at Taiyuan Central Hospital from January 2020 to September 2023.During the study period，406 patients were diagnosed with diabetic foot. After excluding patients with Wagner stage 2 and below, cancer and patients with incomplete data. And because the study population was patients with diabetes-related foot ulcers, other inflammatory causes of foot breakdown such as Buerger's disease, inflammatory bowel disease, and rheumatic diseases in diabetic patients were also excluded. A total of 301 patients with Wagner stage 3 or higher diabetes-related foot ulcers combined with moderate to severe infections were included in the research (Fig 1).**Fig. 1 Flowchart of the patient selection process.**

**Comment 3:**Section 2.2 - why were other comorbid conditions which may influence CRP levels not included as covariates? e.g. other infections, autoimmune or inflammatory conditions? This needs to be added to a section on limitations of the study

**Response 3:**

We think this is an excellent suggestion. We have been mentioned in the limitations of the study as below. Page 13, line 168-170.

First, although we excluded some inflammatory conditions that may affect CRP levels, we cannot exclude the possibility that CRP may be affected by other inflammatory conditions.

**Comment 4:** p.4, paragraph before results - I'd suggest changing the wording of 'effect of CRP' as I don't believe the CRP effects DFI severity, instead it is associated with DFI severity?

**Response 4:**

Thank you for the detailed review. This is an especially important issue. The main finding of our study is also that CRP is associated with DFI severity in result section.Page 10.

**Comment 5:**add very wide confidence intervals to the limitations section

**Response 5:**

We think this is an excellent suggestion. We have made additions to the limitations section. Page 13-14, line 171-173 .

Finally, the wide 95% confidence intervals for the correlation between CRP and DFI severity may be related to sample size, population heterogeneity, and the non-specific nature of CRP as a marker of inflammation, necessitating larger standardised studies with multi-biomarker validation to improve clinical interpretability.

**Comment 6:**the article needs a language and grammar edit, e.g. first line of introduction 'million grown-up? do you mean adults?

**Response 6:**

Thank you again for your valuable suggestions to improve the quality of our manuscript. The first line of the introduction "million adults" means adults. We have corrected the sentence to read "In 2021, the International Diabetes Federation(IDF) notified that over 536.6 million adults global". Page 4, line 2.

**Comment 7:**it is preferred to use the term diabetes-related foot ulcers, rather than diabetic foot ulcer.

**Response 7:**

Thank you for your valuable suggestions. This is a particularly important issue. Based on your suggestions, we have changed the term "diabetic foot ulcer" to " diabetes -related foot ulcers" in our manuscript.

References

1.	Qu YD, Ou SJ, Zhang W, Li JX, Xia CL, Yang Y, et al. Microbiological profile of diabetic foot infections in China and worldwide: A 20-year systematic review. Front Endocrinol. 2024;15:1368046.

2.	Todorova AS, Dimova RB, Chakarova NY, Serdarova MS, Grozeva GG, Georgiev GK, et al. Comparative evaluation of the diagnostic value of procalcitonin and hsCRP for the presence of mild-to-moderate diabetic foot infections. Int J Low Extrem Wounds. 2023 Jun;22(2):353–9.

3.	van Asten SA, Jupiter DC, Mithani M, La Fontaine J, Davis KE, Lavery LA. Erythrocyte sedimentation rate and C-reactive protein to monitor treatment outcomes in diabetic foot osteomyelitis. Int Wound J. 2017 Feb;14(1):142–8.

4.	Qiao L, Yuan H. Prognostic value of C-reactive protein levels in pulmonary infections: A systematic review and meta-analysis. Medicine (Baltimore). 2025 Mar 21;104(12):e41722.

5.	Senneville É, Albalawi Z, van Asten SA, Abbas ZG, Allison G, Aragón-Sánchez J, et al. IWGDF/IDSA guidelines on the diagnosis and treatment of diabetes-related foot infections (IWGDF/IDSA 2023). Diabetes Metab Res Rev. 2024 Mar;40(3):e3687.

6.	Sen P, Demirdal T. Predictive ability of LRINEC score in the prediction of limb loss and mortality in diabetic foot infection. Diagn Microbiol Infect Dis. 2021 May;100(1):115323.

7.	Wukich DK, Hobizal KB, Raspovic KM, Rosario BL. SIRS is valid in discriminating between severe and moderate diabetic foot infections. Diabetes Care. 2013 Nov;36(11):3706–11.

8.	Metineren H, Dülgeroğlu TC. Comparison of the Neutrophil/Lymphocyte Ratio and C-Reactive Protein Levels in Patients With Amputation for Diabetic Foot Ulcers. Int J Low Extrem Wounds. 2017 Mar;16(1):23–8.

9.	Aragón-Sánchez J, Víquez-Molina G, López-Valverde ME, Aragón-Hernández J, Rojas-Bonilla JM, Murillo-Vargas C. Clinical, microbiological and inflammatory markers of severe diabetic foot infections. Diabet Med J Br Diabet Assoc. 2021 Oct;38(10):e14648.

10.	Sun H, Saeedi P, Karuranga S, Pinkepank M, Ogurtsova K, Duncan BB, et al. IDF Diabetes Atlas: Global, regional and country-level diabetes prevalence estimates for 2021 and projections for 2045. Diabetes Res Clin Pract. 2022 Jan;183:109119.

11.	Zhang WQ, Tang W, Hu SQ, Fu XL, Wu H, Shen WQ, et al. C-reactive protein and diabetic foot ulcer infections: A meta-analysis. J Tissue Viability. 2022 Aug;31(3):537–43.

12.	Vitale M, Orsi E, Solini A, Garofolo M, Resi V, Bonora E, et al. Independent association of history of diabetic foot with all-cause mortality in patients with type 2 diabetes: the Renal Insufficiency And Cardiovascular Events (RIACE) Italian Multicenter Study. Cardiovasc Diabetol. 2024 Jan 13;23(1):34.

13.	Liu Y, Zeng S, Ji W, Yao H, Lin L, Cui H, et al. Emerging Theranostic Nanomaterials in Diabetes and Its Complications. Adv Sci Weinh Baden-Wurtt Ger. 2022 Jan;9(3):e2102466.

14.	Capanema NSV, Mansur AAP, Carvalho SM, Martins T, Gonçalves MS, Andrade RS, et al. Nanosilver-Functionalized Hybrid Hydrogels of Carboxymethyl Cellulose/Poly(Vinyl Alcohol) with Antibacterial Activity for Prevention and Therapy of Infections of Diabetic Chronic Wounds. Polymers. 2023 Nov 27;15(23).

15.	Park JH, Suh DH, Kim HJ, Lee YI, Kwak IH, Choi GW. Role of procalcitonin in infected diabetic foot ulcer. Diabetes Res Clin Pract. 2017 Jun;128:51–7.

16.	Zakariah NA, Bajuri MY, Hassan R, Ismail Z, Md Mansor M, Othman H, et al. Is procalcitonin more superior to hs-CRP in the diagnosis of infection in diabetic foot ulcer? Malays J Pathol. 2020 Apr;42(1):77–84.

17.	Gabay C, Kushner I. Acute-phase proteins and other systemic responses to inflammation. N Engl J Med. 1999 Feb 11;340(6):448–54.

18.	McFadyen JD, Kiefer J, Braig D, Loseff-Silver J, Potempa LA, Eisenhardt SU, et al. Dissociation of C-reactive protein localizes and amplifies inflammation: Evidence for a direct biological role of C-reactive protein and its conformational changes. Front Immunol. 2018;9:1351.

19.	Zakariah NA, Bajuri MY, Hassan R, Ismail Z, Md Mansor M, Othman H, et al. Is procalcitonin more superior to hs-CRP in the diagnosis of infection in diabetic foot ulcer? Malays J Pathol. 2020 Apr;42(1):77–84.

20.	Senneville É, Albalawi Z, van Asten SA, Abbas ZG, Allison G, Aragón-Sánchez J, et al. IWGDF/IDSA Guidelines on the Diagnosis and Treatment of Diabetes-related Foot Infections (IWGDF/IDSA 2023). Clin Infect Dis Off Publ Infect Dis Soc Am. 2024 Mar;40(3):e3687.
